# Auranofin radiosensitizes tumor cells through targeting thioredoxin reductase and resulting overproduction of reactive oxygen species

**DOI:** 10.18632/oncotarget.16113

**Published:** 2017-03-10

**Authors:** Hui Wang, Soumaya Bouzakoura, Sven de Mey, Heng Jiang, Kalun Law, Inès Dufait, Cyril Corbet, Valeri Verovski, Thierry Gevaert, Olivier Feron, Dirk Van den Berge, Guy Storme, Mark De Ridder

**Affiliations:** ^1^ Department of Radiotherapy, Universitair Ziekenhuis Brussel, Vrije Universiteit Brussel, Brussels, Belgium; ^2^ Laboratory of Molecular and Cellular Therapy, Vrije Universiteit Brussel, Brussels, Belgium; ^3^ Pole of Pharmacology and Therapeutics (FATH), Institut de Recherche Expérimentale et Clinique (IREC), Université Catholique de Louvain, Brussels, Belgium

**Keywords:** auranofin, radiosensitization, ROS, thioredoxin reductase, buthionine sulfoximine

## Abstract

Auranofin (AF) is an anti-arthritic drug considered for combined chemotherapy due to its ability to impair the redox homeostasis in tumor cells. In this study, we asked whether AF may in addition radiosensitize tumor cells by targeting thioredoxin reductase (TrxR), a critical enzyme in the antioxidant defense system operating through the reductive protein thioredoxin. Our principal findings in murine 4T1 and EMT6 tumor cells are that AF at 3–10 μM is a potent radiosensitizer *in vitro*, and that at least two mechanisms are involved in TrxR-mediated radiosensitization. The first one is linked to an oxidative stress, as scavenging of reactive oxygen species (ROS) by N-acetyl cysteine counteracted radiosensitization. We also observed a decrease in mitochondrial oxygen consumption with spared oxygen acting as a radiosensitizer under hypoxic conditions. Overall, radiosensitization was accompanied by ROS overproduction, mitochondrial dysfunction, DNA damage and apoptosis, a common mechanism underlying both cytotoxic and antitumor effects of AF. In tumor-bearing mice, a simultaneous disruption of the thioredoxin and glutathione systems by the combination of AF and buthionine sulfoximine was shown to significantly improve tumor radioresponse. In conclusion, our findings illuminate TrxR in cancer cells as an exploitable radiobiological target and warrant further validation of AF in combination with radiotherapy.

## INTRODUCTION

The role of reactive oxygen species (ROS) in tumor biology and therapy is currently evolving in two directions. First, ROS clearly sustain tumor progression and the acquisition of chemo- and radioresistance, which are conferred by activation of the antioxidant defense systems that allows tumor cells to proliferate under chronic oxidative stress [1]. Indeed, the level of glutathione (GSH) and the reductive protein thioredoxin (Trx), which maintain the redox homeostasis by eliminating ROS, is frequently increased in human malignancies and linked to poor prognosis [2, 3]. Given an elevated intrinsic ability of tumor cells to deal with oxidative damage, the antioxidant approach based on dedicated ROS scavengers, like N-acetyl cysteine (NAC) and mitoTEMPO, has so far not found a place in clinical practice yet suggests interesting possibilities to prevent tumor metastasis [4]. On the other hand, the overproduction (rather than scavenging) of ROS is known to kill tumor cells, a mechanism behind the antitumor effect of mitomycin C, doxorubicin and ionizing radiation [5, 6]. The latter pro-oxidant strategy is now under intensive development with a focus on gamma-glutamyl cysteine synthase (γ-GCS) and Trx reductase (TrxR), as these enzymes are critical in the biosynthesis of GSH and Trx and thus represent promising cancer targets [1].

GSH forms the main intracellular component involved in a redox balance and is essential for cell proliferation and cell cycle progression [7]. In addition, GSH determines the storage of intracellular cysteine and eventually regulates the functional status of redox sensitive and cysteine-dependent transcription factors, such as NF-κB, relevant to both inflammation and apoptosis. Therefore, major efforts in the past have been already aimed at depleting GSH by buthionine sulfoximine (BSO), a potent irreversible inhibitor of γ-GCS [1, 8]. This approach appeared to be efficient in sensitizing tumor cells to several chemotherapeutic drugs and radiation [9, 10], and its combination with melphalan is now under clinical investigation (https://clinicaltrials.gov/ct2/show/NCT00005835).

The history of TrxR is linked to the anti-arthritic drug auranofin (AF), a gold complex with oxidative state (I), which can elicit strong cytotoxicity against tumor cells through the overproduction of ROS that in turn triggers the apoptotic pathway [11–16]. The primary molecular targets of AF are described to be mitochondrial and (to a lesser extent) cytoplasmic TrxR [17], although other mechanisms at the level of proteasome may also contribute to apoptosis [18]. An intriguing finding is that AF may overcome cisplatin resistance, since it impairs mitochondria rather than DNA [19]. Overall, despite being an established anti-inflammatory drug, AF seems to offer great promise in the context of pro-oxidant cancer therapy, and is considered for the combined modality treatment of leukemia (https://clinicaltrials.gov/ct2/show/NCT01419691), lung cancer (https://clinicaltrials.gov/ct2/show/NCT01737502) and epithelial ovarian cancer (https://clinicaltrials.gov/ct2/show/NCT01747798).

Recently, the antitumor effects of AF have been explored in more detail in preclinical models since this drug is safe for cancer patients and fits the concept of drug repurposing. AF induced strong cytotoxicity in human chronic leukemia and gastric cancer cells due to a lethal endoplasmic reticulum stress and mitochondrial dysfunction accompanied by ROS overproduction [11, 12]. This effect could be potentiated by other ROS inducers resulting in an increased apoptosis of tumor cells and translated into a meaningful growth inhibition of tumor xenografts [12]. Next to a direct antitumor effect, AF was able to suppress the outgrowth of pulmonary metastases in a model of human osteosarcoma in nude mice, and the inhibition of metastatic phenotype was explained by ROS-dependent apoptosis [13]. Finally, AF was confirmed to be effective in drug-resistant multiple myeloma and chronic leukemia cells, wherein triggering of apoptosis by alternative ROS-dependent and independent mechanisms have been elucidated in depth [14, 15]. To the best of our knowledge, only one paper so far showed that AF can enhance the radiation response in tumor cells even though the disruption of antioxidant defense systems is a long-standing concept for radiosensitization [20].

In this study, we examined the radiosensitizing potential of AF *in vitro* with regard to its plasma concentrations and further validated radiotherapeutic applications in tumor-bearing mice. We found that the inhibition of TrxR and resulting ROS overproduction is the principal mechanism of tumor cell radiosensitization, which could be significantly enhanced by GSH depletion. Therefore, our findings suggest the TrxR/Trx system as a promising radiobiological target and prompt further evaluation of AF for radiosensitizing purposes.

## RESULTS

### AF caused apoptosis and cytotoxicity in mouse tumor cells

**Figure 1:**
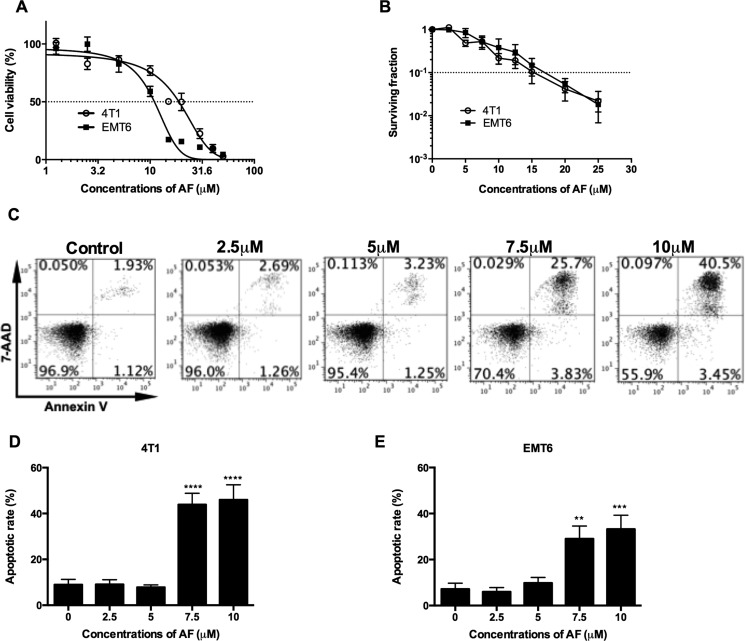
AF caused apoptosis and cytotoxicity in mouse tumor cells (**A**) Tumor cells were treated with AF for 2 h with indicated concentrations, and one day later analyzed for cell viability by MTT assay. Data are shown as mean ± SD (*n* = 3). (**B**) Following the same treatments, cell viability was analyzed by an 8-day colony formation assay. Data are shown as mean ± SD (*n* = 3). (**C**) Representative scatter plots of apoptosis in 4T1 cells, after AnnexinV/7-AAD staining and assessment by flow cytometry. (**D**) Summarized data on AF-induced apoptosis in 4T1 and (**E**) EMT6 tumor cells. Data are shown as mean ± SD (*n* ≥ 3). One-way ANOVA with Bonferonni's multiple comparison test was used to calculate statistics: *p < 0.05, **p < 0.01, ***p < 0.001, ****p < 0.0001.

Our preclinical models are based mainly on EMT6 and 4T1 mouse mammary carcinoma cell lines and tumors, which have been extensively studied in our lab for hypoxic radiosensitization and immunological profiling [21, 22]. To investigate the cytotoxic properties of AF, EMT6 and 4T1 cell cultures were treated for 2 h and cell viability was determined by MTT and colony formation assays (Figure 1A and 1B). In a short-term (2 days) MTT assay, AF decreased the cell viability in a dose-dependent manner with the IC*^50^* values of 19 and 11 μM for 4T1 and EMT6 cells respectively. In a long-term (8 days) clonogenic assay, a survival fraction (SF) of 0.1 was detected at 15 and 17 μM respectively, indicating that concentrations below 10 μM produce less than 1 log cell kill and are suitable for radiosensitization. To determine whether apoptosis was involved in AF-induced cytotoxicity, the exposed tumor cells were stained with Annexin-V/7-AAD followed by flow cytometry analysis in 4T1 cells (Figure 1C–1D), and in EMT6 cells (Figure 1E and Supplementary Figure 2A). At 10 μM, the apoptotic rates in 4T1 and EMT6 cells were respectively 46% and 33%, which mainly reflected late apoptosis in 7-AAD-positive tumor cells. Thus, other than apoptotic death pathways contributed to AF-induced cytotoxicity in EMT6 and 4T1 cells as well.

### AF inhibited TrxR and triggered ROS overproduction

**Figure 2:**
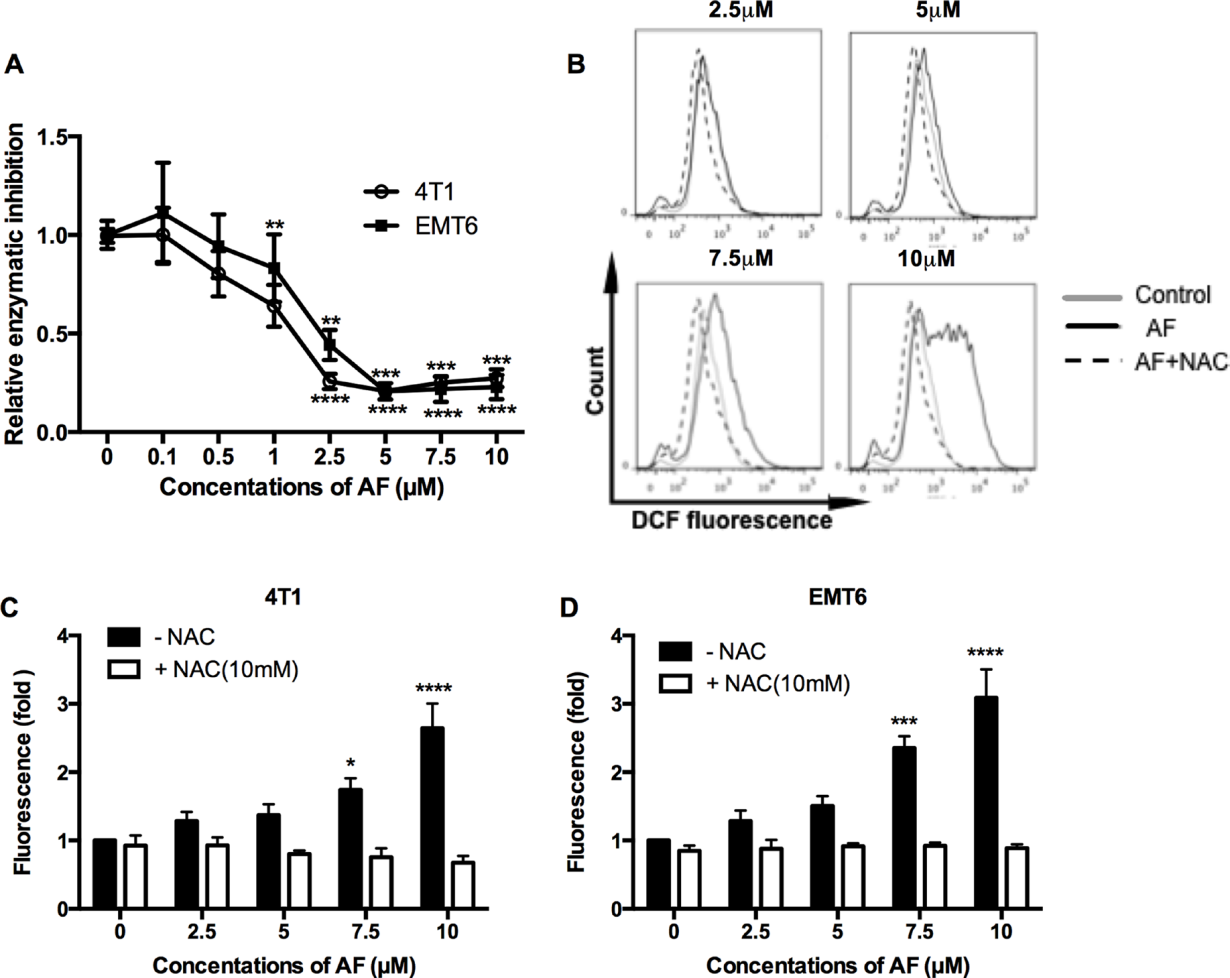
AF inhibited TrxR and triggered ROS overproduction in tumor cells (**A**) TrxR activity was measured by commercial kit and all values were normalized to untreated controls. Data are shown as mean ± SD (*n* ≥ 3). (**B**) Representative histogram of intracellular ROS in 4T1 cells, as analyzed by flow cytometry using the CM-H*^2^*DCFDA probe. (**C**–**D**) Summarized data on ROS production in 4T1 and EMT6 cells pretreated with the ROS scavenger NAC (10 mM) prior to AF. Data are shown as mean ± SD (*n* = 3). One-way ANOVA with Bonferonni's multiple comparison test was used to calculate statistics: *p < 0.05, **p < 0.01, ***p < 0.001, ****p < 0.0001.

It is well accepted that AF elicits cytotoxicity mainly due to its inhibitory effect on TrxR resulting in an overload ROS [23, 24]. Therefore, first, we assessed the ability of AF to inhibit TrxR activity in 4T1 and EMT6 cells (Figure 2A). The inhibitory effects in both cell lines were evident above 1 μM with a profound inactivation of TrxR at 5–10 μM (Figure 2A). Next, the intracellular redox status was evaluated through ROS generation using the fluorescent probe CM-H*^2^*DCFDA. As shown in Figure 2B–2D (and Supplementary Figure 2B), ROS production was induced in a dose-dependent manner and was significantly upregulated at 7.5 (*p < 0.05 and ***p < 0.001) and 10 μM (****p < 0.0001) in 4T1 and EMT6 cells respectively, according to the shift in DCFDA signal. Apparently, the inhibition of TrxR by AF occurs at relatively lower concentrations than those causing a considerable increase in ROS, suggesting that other antioxidative systems (such as GSH) may display compensatory effects. Finally, the importance of ROS production in observed effects was confirmed by using NAC, a thiol-reducing antioxidant agent. Pretreatment of 4T1 and EMT6 cells with NAC for 1 h at 10 mM effectively attenuated the ROS overproduction caused by AF, as shown in Figure 2B–2D further detailed in Supplementary Figure 2B. In addition, NAC counteracted AF-induced cytotoxicity (data not shown), indicating that ROS production and cytotoxicity are linked.

### AF radiosensitized aerobic tumor cells

**Figure 3:**
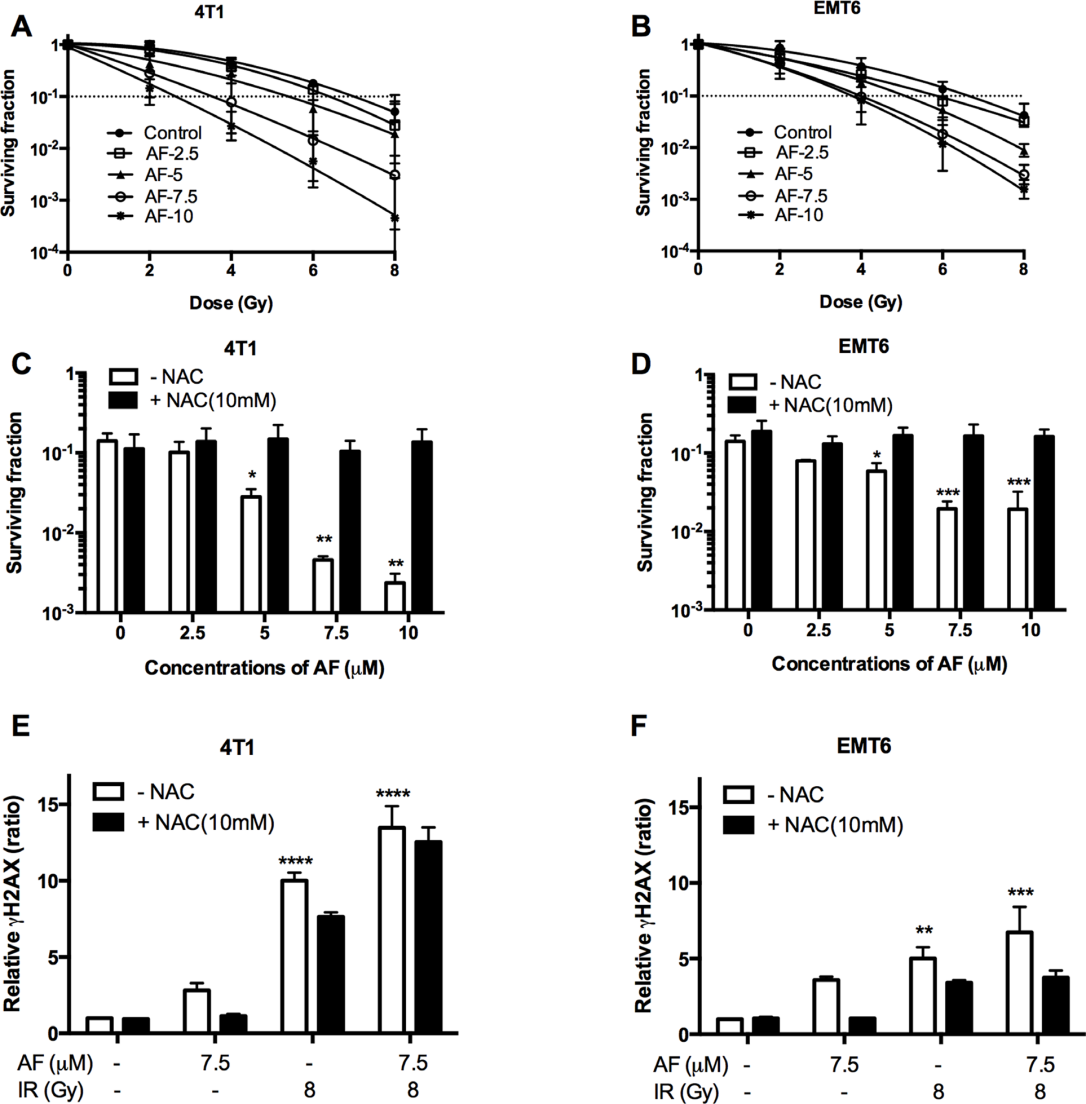
AF radiosensitized aerobic tumor cells and enhanced radiation induced DNA damage 4T1 and EMT6 cells were treated with AF for 2 h at indicated concentrations, while NAC (10 mM) was added 1 h prior and during treatment. (**A**–**B**) The radiosensitizing effect of AF was assessed by colony formation assay. Data are shown as mean ± SD (*n* = 3). (**C**–**D**) Counteracting effect of the ROS scavenger NAC at 6 Gy. (**E–F**) Double-strand DNA breaks were analyzed by flow cytometry using the γH2AX-based foci measurements. Data are shown as mean ± SD (*n ≥* 3). One-way ANOVA with Bonferonni's multiple comparison test was used to calculate statistics: *p < 0.05, **p < 0.01, ***p < 0.001, ****p < 0.0001.

The radiosensitizing potential of AF was examined at concentrations below 10 μM, which are sub-cytotoxic (< 1 log cell kill, Figure 1B) in 4T1 and EMT6 cells. Based on this, tumor cells were treated with AF at 2.5, 5, 7.5, and 10 μM for 2 h and subsequently exposed to various radiation doses under aerobic conditions (Figure 3A–3B). In line with an increased ROS production shown in Figure 2C–2D, we found a dose-dependent radiosensitization with an enhancement ratio above 2 at 7.5–1 0 μM, which showed a synergism of AF and radiation. To confirm the role of ROS in AF-induced radiosensitization (at 6 Gy), we again applied NAC pretreatment that fully reversed radiosensitization in both 4T1 and EMT6 cells (Figure 3C–3D). Since AF is known to induce ROS-mediated DNA damage, a fundamental mechanism behind radiation-induced cell death, we next examined double-strand DNA breaks by quantifying the phosphorylation status of γH2AX. Radiation (8 Gy) or AF (7.5 μM) alone increased the number of γH2AX foci, which were suppressed by NAC in both cell lines (Figure 3E–3F). Combined treatment displayed an additive effect and increased DNA damage by more than 7-times compared with control (**p < 0.01, ***p < 0.001 and ****p < 0.0001). Taken together, these data indicate a mechanistic link between radiosensitization, DNA damage and ROS overproduction induced by AF through TrxR inactivation.

### AF radiosensitized hypoxic tumor cells

**Figure 4:**
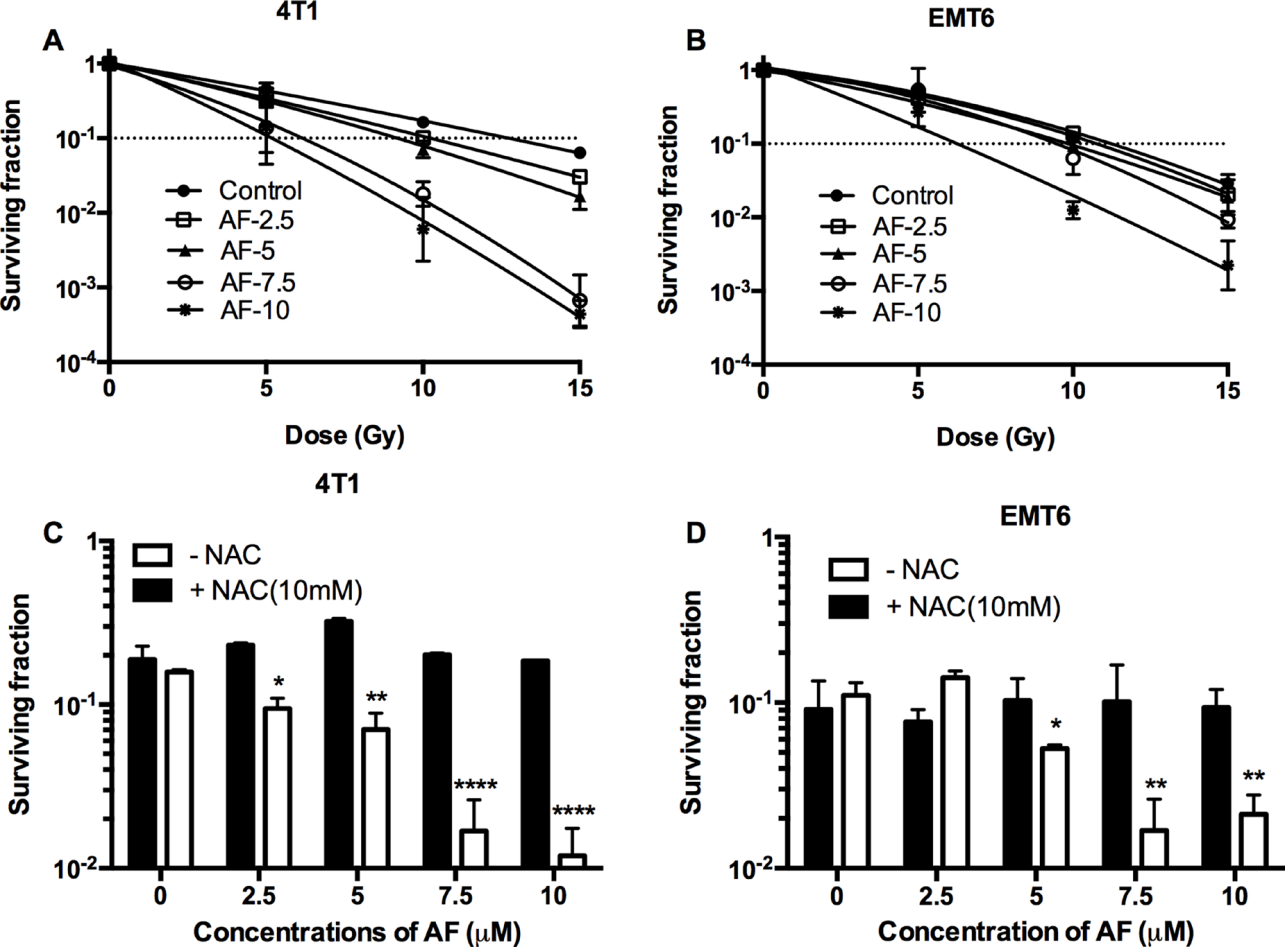
AF radiosensitized hypoxic tumor cells 4T1 cells were treated with AF for 2 h at indicated concentrations, while NAC (10 mM) was added 1 h prior and during treatment. To assess hypoxic radiosensitivity, subconfluent cultures were irradiated in a metabolic hypoxia model TMCS. (**A**–**B**) Radiosensitizing effect of AF was assessed by colony formation assay. Data are shown as mean ± SD (*n* = 3). (**C**–**D**) Counteracting effect of the ROS scavenger NAC at 10 Gy. Data are shown as mean ± SD (*n* ≥ 3). One-way ANOVA with Bonferonni's multiple comparison test was used to calculate statistics: *p < 0.05, **p < 0.01, ***p < 0.001, ****p < 0.0001.

Since tumor hypoxia is known to be radioprotective and causes therapeutic failure, we assessed the radiosensitizing potential of AF in a tissue-mimetic culture system (TMCS), a metabolic hypoxia model, described in detail by our laboratory in previous studies [22]. First, in murine 4T1 and EMT6 tumor cells, we observed an impaired hypoxic radiosensitivity when compared with aerobic survival curves. Indeed, a 1 log cell kill (SF = 0.1) in hypoxia was achieved with 12.5 and 11 Gy in 4T1 and EMT6 cells (Figure 4A–4B), while under aerobic conditions the same effect was observed at 7 and 6.6 Gy (Figure 3A–3B). Next, a clear AF-induced radiosensitization was observed at 7.5–1 0 μM with an enhancement ratio up to 2.5 and 1.8 for 4T1 and EMT6 cells respectively, pointing to a superior effect in more radioresistant 4T1 cells (Figure 4A–4B). This effect (at 10 μM) was reversed by the addition of NAC in both cell lines (Figure 4C–4D). Afterwards, we validated the radiosensitizing potential of AF in human HCT116 colorectal cancer cells. Under hypoxic conditions, AF induced a dose-dependent radiosensitization with an up to 2.1-fold enhanced radioresponse after exposure to 10 μM AF (Supplementary Figure 3A). Similar to aerobic tumor cells, AF induced radiosensitization was counteracted by NAC at 10 mM while NAC on its own did not modulate radioresponse in hypoxia (Supplementary Figure 3B). Overall, NAC pretreatment (without AF) did not exert any impact on radioresponse in all tumor cell lines including 4T1, EMT6 and HCT116, as further detailed in Supplementary Figure 4. At this point, we compared the radiosensitivity profiles of EMT6 versus 4T1 tumor cells (Figures 3, 4), and concluded that the latter model displays a trend to decreased intrinsic radiosensitivity. Therefore, the next steps in our study have been limited to 4T1 model assuming that these tumor cells feature a more efficient antioxidant system – an optimal target for AF and BSO.

### AF induced mitochondrial dysfunction in 4T1 tumor cells

**Figure 5:**
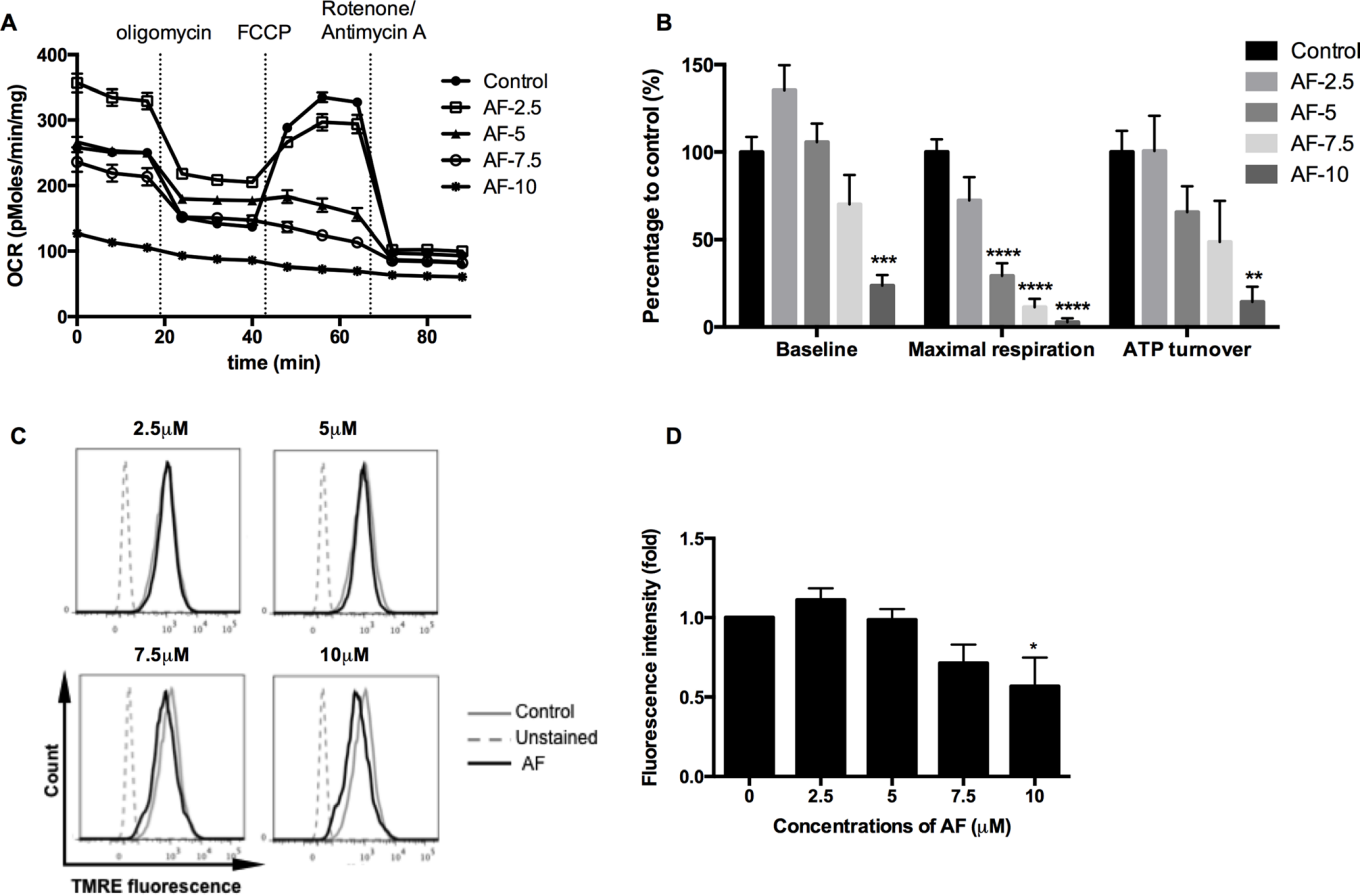
AF induced mitochondrial dysfunction in tumor cells 4T1 cells were treated with AF for 2 h at indicated concentrations and the respiratory profiles were examined by a Seahorse Analyzer. (**A**) Dissection of respiratory rates by the sequential injection of oligomycin, FCCP, rotenone and antimycin A at indicated time points. (**B**) Summarized data on the baseline respiratory rate, maximum respiratory capacity and ATP turnover. Data are shown as mean ± SD (*n* = 5). (**C**–**D**) Representative measurements of ΔΨm in 4T1 cells by flow cytometry and summarized data on membrane potential. Data are shown as mean ± SD (*n* = 3). One-way ANOVA with Bonferonni's multiple comparison test was used to calculate statistics: *p < 0.05, **p < 0.01, ***p < 0.001, ****p < 0.0001.

As ROS generation is a by-pass of mitochondrial bioenergetics, the respiratory status of tumor cells has been further dissected in a Seahorse analyzer using a sequence of specific inhibitors. AF significantly decreased the basal oxygen consumption rate, and in addition the maximal respiratory capacity and ATP output in 4T1 tumor cells (**p < 0.01, ***p < 0.001 and ****p < 0.0001), as illustrated in Figure 5A and further summarized in Figure 5B. Overall, the inhibition of oxygen consumption and the resulting sparing of oxygen, a potent radiosensitizer, seem to offer an additional radiosensitizing effect specifically in hypoxia next to the basic mechanism through ROS overproduction. To get more insight into mitochondrial dysfunction induced by AF, we measured the mitochondrial membrane potential ΔΨm, an important parameter of membrane integrity. We anticipated that membrane depolarization would explain an uncoupled oxidative phosphorylation (ATP decline) and resulting apoptosis induced by AF in 4T1 tumor cells (Figures 5A–5B and 1C–1D). As demonstrated in Figure 5C–5D (and Supplementary Figure 2C), exposed tumor cells showed a decreased signal of TMRE, a cell permeable fluorescent dye, which is effectively retained in intact but not damaged mitochondria. We observed a dose-dependent decrease of ΔΨm induced by AF with 50% inhibition at 10 μM (*p < 0.05).

### BSO potentiated AF-induced radiosensitization in 4T1 tumor cells

**Figure 6:**
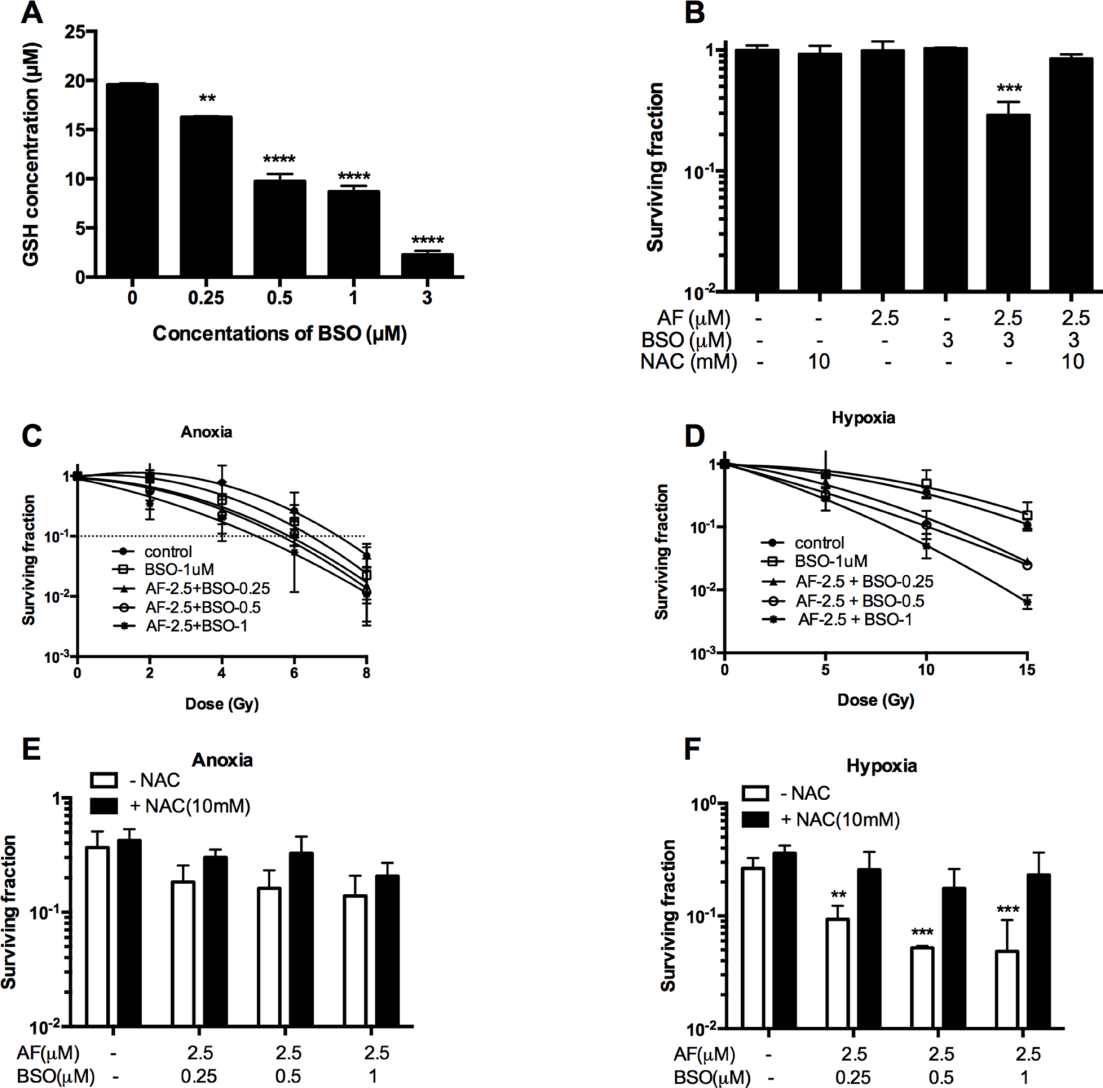
BSO potentiated AF-induced radiosensitization in tumor cells 4T1 cells were exposed to AF and/or BSO at indicated concentrations for 2 and 16 h respectively. (**A**) Depletion of glutathione by BSO in 4T1 cells. Data are shown as mean ± SD (*n ≥* 3). (**B**) Synergistic cytotoxicity of AF combined with BSO applied at non-cytotoxic concentrations. Data are shown as mean ± SD (*n ≥* 3). (**C**–**D**) Aerobic and hypoxic radiosensitization by AF combined with BSO, as measured by colony formation assay. Data are shown as mean ± SD (*n* = 3). (**E**–**F**) Counteracting effect of the ROS scavenger NAC under aerobic and hypoxic conditions respectively. Data are shown as mean ± SD (*n* = 3). One-way ANOVA with Bonferonni's multiple comparison test was used to calculate statistics: *p < 0.05, **p < 0.01, ***p < 0.001, ****p < 0.0001.

Given that GSH may back-up the deficit of reduced Trx, we asked whether a dual targeting of those systems by AF and BSO may be beneficial in terms of cytotoxicity and radiosensitization. First, we assessed GSH biosynthesis in 4T1 cells, and found a progressive depletion of total GSH caused by BSO at 0.25–3 μM (**p < 0.01 and ****p < 0.0001) with a half-decline at 0.5 μM (Figure 6A). Next, we examined the effect of combined AF and BSO at non-cytotoxic concentrations of 2.5 and 3 μM respectively. Strikingly, this combination displayed significant synergism with a resulting SF of 0.3 (***p < 0.001), which was abolished by NAC (Figure 6B). Based on these data, similar combinations were applied for radiosensitizing purposes under both aerobic and hypoxic conditions. BSO (1 μM) and AF (2.5 μM) alone did not alter radiosensitivity, while their combination enhanced aerobic and hypoxic radioresponse by 1.5 and 1.9 times respectively (Figure 6C–6D). Thus, this combination showed a preferential radiosensitizing effect in hypoxic 4T1 cells, which displayed a clear radioprotection in the TMCS, our model of metabolic hypoxia. As expected, those synergistic effects were counteracted by NAC (Figure 6E–6F). The observed synergism between AF and BSO was of particular interest for *in vivo* applications since the plasma-achievable levels of AF are known to be around 3 μM, which is on a low side of effective radiosensitizing concentrations according to our data (Figures 3, 4).

### AF combined with BSO enhanced radioresponse of 4T1 tumor

**Figure 7:**
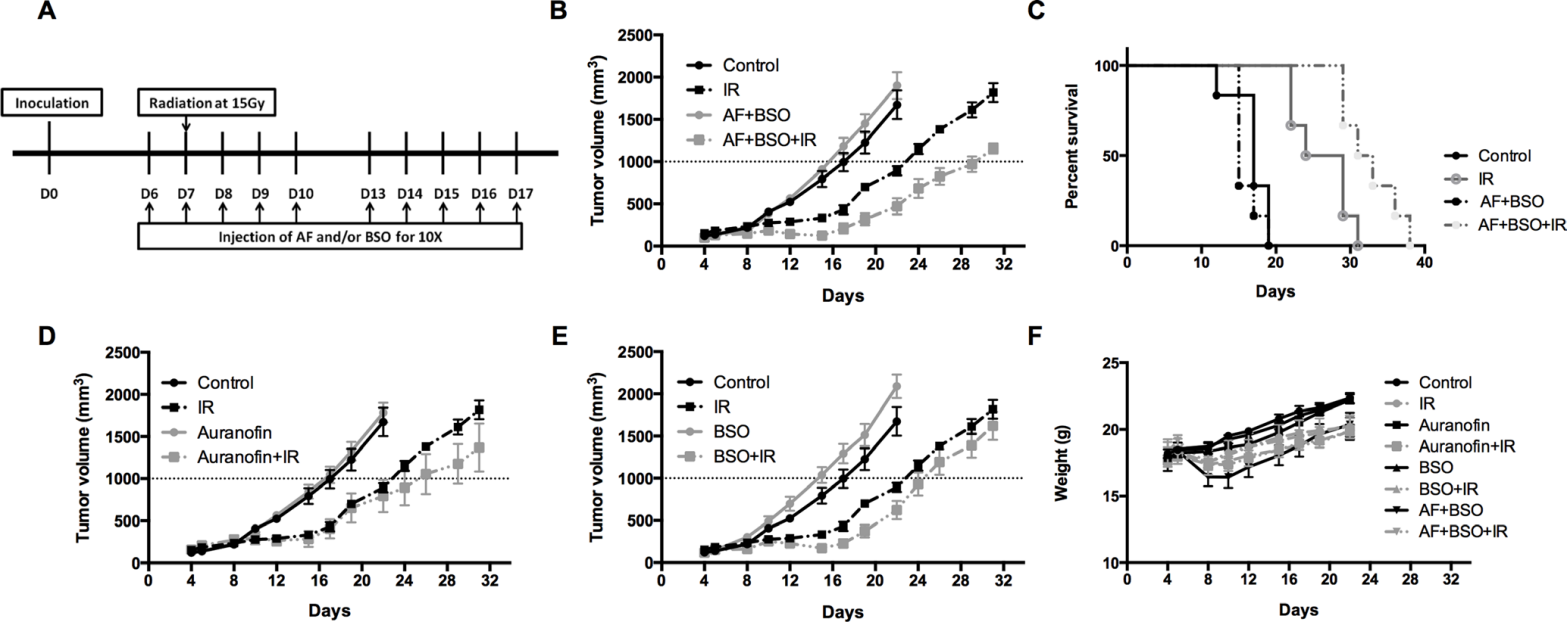
AF combined with BSO enhanced the radioresponse of 4T1 tumor in Balb/c mice AF and BSO were administered subcutaneously for 10 times to tumor-bearing mice, and single dose radiation at 15 Gy was delivered on the second day of treatment. (**A**) Experimental scheme depicting used treatment protocols. (**B**) Tumor growth in mice treated with radiation and the combination of AF (3 mg/kg) and BSO (25 mg/kg). (**C**) Survival curves of mice euthanized at a diameter of 15 mm. (**D**) Tumor growth in mice treated with radiation and AF only. (**E**) Tumor growth in mice treated with radiation and BSO only. (**F**) Assessment of toxicity by body weight loss.

**Figure 8:**
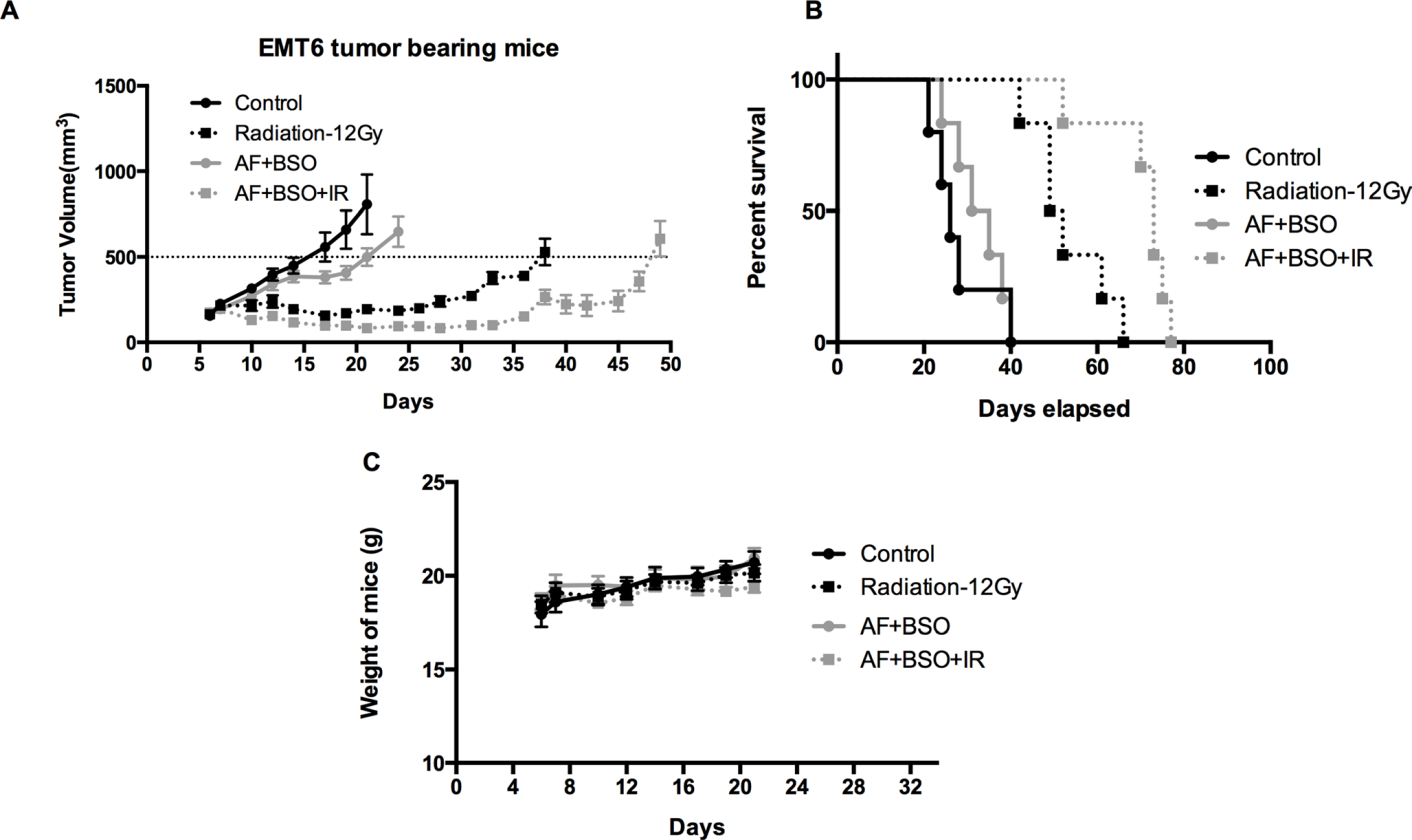
AF combined with BSO enhanced the radioresponse of EMT6 tumor in Balb/c mice AF and BSO were administered as above in 4T1 tumor model, and single dose radiation at 12 Gy was delivered on the second day of treatment. (**A**) Tumor growth of mice treated with radiation and the combination of AF (3 mg/kg) and BSO (25 mg/kg). (**B**) Survival curves of mice euthanized at diameter of 15 mm. (**C**) Assessment of toxicity by body weight loss.

To validate the *in vitro* findings, the radiosensitizing effect of AF combined with BSO was evaluated in both 4T1 and EMT6 tumor-bearing mice. The experimental scheme is depicted in Figure 7A. In 4T1 tumor model, radiation alone at 15 Gy delayed tumor growth by 6 days measured at a tumor volume of 1000 mm^3^ (Figure 7B). AF combined with BSO enhanced tumor radioresponse resulting in a tumor growth delay of 13 days and thereby significantly increased the medium survival rate (Figure 7B and 7C), while neither of these pharmaceuticals were effective when administered on their own (Figure 7D and 7E). Importantly, BSO (25 mg/kg) and/or AF (3 mg/kg) applied for 10 days were safe and represented the maximal tolerated doses with no significant body weight loss (Figure 7F). In EMT6 tumor model, which is more radiosensitive than 4T1, radiation alone at 12 Gy delayed tumor growth by 20 days measured at a tumor volume of 500 mm^3^ (Figure 8A). AF plus BSO further delayed the tumor growth by 9 days compared with radiation alone (Figure 8A), and this combination once again increased the survival rate of tumor-bearing mice (Figure 8B). Of note, similar to 4T1 tumor model, BSO plus AF treatment did not show notable toxicity in mice, as measured by the body weight loss (Figure 8C). Altogether, our data point to the necessity of dual targeting of the TrxR/GSH systems by the combination of AF and BSO.

## DISCUSSION

In this study we examined the hypothesis that the anti-arthritic drug AF may be repurposed for radiotherapeutic applications and undertook the first step to evaluate its radiosensitizing potential in mouse 4T1 and EMT6 tumor cells. Our fundamental findings are that AF at 3–10 μM increases tumor cell radiosensitivity *in vitro* over 2-fold, and two mechanisms appear to be engaged. The first one is tightly related to an oxidative stress, as the classic scavenger NAC abolished both ROS overproduction and radiosensitization yet by itself did not modulate radioresponse. These divergent properties may be explained by its ability to mitigate radiation-induced DNA damage but not affect the cell kill [25]. The latter result is further corroborated by the very efficient TrxR/GSH anti-oxidant systems in tumor cells that overweigh the scavenging potential of NAC under basal levels of ROS in the absence of AF. Under tumor-modeling hypoxic conditions, we also observed the arrest of oxygen consumption in mitochondria suggesting an alternative effect of AF through spared oxygen, a potent radiosensitizer. The latter mechanism can be detected only in metabolically induced hypoxia, which recently attracted more attention given that the inhibition of mitochondrial respiration rather than an increase in oxygen supply may offer an efficient way to overcome hypoxic radioresistance [22, 26]. Tumor hypoxia is a recognized negative factor for therapy outcomes, and the ability of AF to reverse metabolic radioresistance suggests the promising possibility to preferentially target poorly oxygenated tumor cells.

It is worthy to stress that the nature of effects so far ascribed to AF, and more generally to gold formulations, is multifaceted and continues to expand. The metallic gold in a form of nanoparticles is non-toxic up to 1000 μM and displays radiosensitizing properties due to the secondary low-energy beams (Auger electrons) that amplify radiation-induced DNA damage [27]. Both low toxicity and direct radiosensitization are lost once the gold atom is oxidized to a complex (I), with AF as an example, and further to a more stable complex (III), both of which were broadly screened for cytotoxicity and antitumor effects [28]. AF was reported to inactivate TrxR above 3 μM that in part explains strong cytotoxicity against tumor cells, as lack of this redox-critical enzyme results in mitochondrial dysfunction caused by ROS [17].

In line, we found (i) dose-dependent TrxR inhibition, (ii) followed by ROS overproduction and (iii) accompanied by mitochondrial and further DNA damage. This sequence of events caused by AF has been described in many types of tumor cells undergoing apoptosis [11, 12, 16], which was detected in our model as well yet leaving a space for other (unidentified) death pathways. In our settings, apoptosis was detected above 5 μM AF in a threshold-like manner, a phenomenon that seems to be triggered once the level of ROS becomes incompatible with the mitochondrial membrane integrity [29]. Interestingly, AF-induced radiosensitization through TrxR occurs at 1–2 log lower concentrations when compared with that of bio-inert metallic gold operating through photoelectrons [30]. More importantly, our data indicate that AF at plasma relevant levels is exploitable for radiosensitization, a novel mechanism that points to an appealing opportunity of rescaling current clinical trials so far limited to chemotherapeutic goals (https://clinicaltrials.gov/ct2/show/NCT01747798, https://clinicaltrials.gov/ct2/show/NCT01737502). Supporting this conclusion, two other gold (I) complexes, namely Au(SCN)(PEt(3)) and an Au(I)-indole, were reported to possess radiosensitizing properties as well [31, 32].

It is essential to acknowledge that ROS-mediated effects are complex and interplay with the multiple inflammatory pathways associated with tumor progression, as schematically summarized in Supplementary Figure 1. Briefly, ROS may display both pro- and anti-tumor properties, and on the top we could expect both pro- and anti-inflammatory effects from AF, considering the history of its use as an anti-arthritic drug [33]. The latter medication was linked to the inhibition of pro-inflammatory mediators (IL-6, IL-1β, TNFα, NF-κB etc.) and oxidative burst in monocytes and granulocytes respectively [34, 35]. This picture is opposed to a more recent pro-oxidant approach, where drug-induced ROS cause apoptosis/cytotoxicity in tumor cells [1]. It remains to be clarified whether an oxidative stress signature is of prognostic value, while the inflammatory desmoplastic reaction in tumor-associated fibroblasts is known to promote tumorigenesis [36]. In this context, our findings are obviously valid for tumor cells only and should not be projected to the host stromal and immune cells involved in cancer-related inflammation. However, we previously showed that myeloid cells can undergo pro-tumor polarization [37], accompanied by an increased neutrophil-to-lymphocyte ratio and arginase activation [21], in line with the recent literature [38, 39] and the extensive background on the immune landscape in cancer [40, 41]. Hence, AF might target the myeloid lineage differently from tumor cells and thereby offer still another way to restore tumor radioresponse through immune cells, as we previously proposed for rectal cancer [42].

While this speculative mechanism remains to be explored, our preliminary data suggest that the *in vitro* radiosensitizing effect of AF, evident in aerobic and hypoxic 4T1 and EMT6 tumor cells, is a probable cause of an improved tumor radioresponse *in vivo*. This setting was designed with regard to a current consensus on the coordinated role of the γ-GCS/GSH and TrxR/Trx antioxidant systems in clinical chemo- and radioresistance, as overviewed in detail elsewhere [9, 19, 43]. The γ-GCS inhibitor BSO is a well-documented chemo- and radiosensitizer in experimental models [10], and in our hands it potentiated the cytotoxic and radiosensitizing effect of AF *in vitro*. The combination AF plus BSO showed a preferential radiosensitizing effect in hypoxic tumor cells, which featured an impaired radioresponse in the TMCS, our model of metabolic hypoxia. In mice, their simultaneous administration enhanced radioresponse in EMT6 and 4T1 mammary carcinomas, in line with a very recent report in a MDA-MB-231 breast cancer model [20]. We concluded that the combination of AF and BSO could be a promising radiosensitizing strategy justified in view of them being ready-to-use pharmaceuticals for clinical evaluation.

In contrast to some other reports, we were not able to detect the antitumor effect induced by AF and BSO alone but confirmed low toxicity associated with their chronic use. A plausible explanation is that radiosensitization may occur at lower sub-cytotoxic drug levels (< 1 log cell kill) than those required for the direct inhibition of fast growing tumors, therefore pointing to a synergistic interaction with irradiation. Clearly, a plasma achievable level of 3 μM, known for AF [44], is a limiting factor of cytotoxicity and radiosensitization *in vivo*, which are below the observed effects in cell cultures rescaled to 5–10 μM. Our preclinical settings also suffer from some limitations due to the intramuscular tumor site, therefore lacking the physiological microenvironment in the mammary gland. However, radiotherapeutic applications in mice require a practical way to immobilize the bulky tumors precisely within the irradiation field, and are generally based on non-orthotopic implantations [20]. On the other hand, our pharmacological treatments do reflect clinical trials, where AF is administered chronically at safe maximal tolerated doses.

Likewise, recent preclinical studies highlighted the chemotherapeutic importance of knocking down the antioxidant cellular defense by AF, and described an impressive list of divergent ROS-mediated mechanisms, which suppressed the growth of local tumors [11, 12] and metastasis [13] or reversed chemoresistance [19]. Along with improved radiation responses, AF sensitized breast cancer stem cells, and in combination with BSO decreased cell migration and invasion [20]. Of note, inhibition of TrxR by curcumin and 1, 2, 5-selenadiazole showed a comparable array of antitumor effects including radiosensitization [32, 45–47], while the disruption of GSH pathways by genistein and gadolinium (III) texaphyrin resulted in ROS-mediated radiosensitization [48, 49]. Compelling evidence also suggests that the abundant GSH pool is an important backup to keep Trx reduced, and therefore a dual targeting of the GSH/Trx antioxidant systems is required [43, 50].

The next logical development is to apply a triple inhibition of GSH/Trx/Nrf2, where the latter mediator is responsible for maintaining the anti-oxidant system in a reduced state [51]. Blocking the glycolysis and pentose cycle pathways may also interrupt GSH/Trx-dependent cellular defense, while NAC reverses an amplified clonogenic cell death triggered by ROS [52]. Both Trx and GSH are currently recognized as key targets in chemo/radiosensitizing strategies in line with our data, while a single TrxR inactivation may not be always enough to achieve meaningful effects. Indeed, genetic knocking-down of TrxR by siRNA appears to be short of efficiency to inhibit its enzymatic activity or to provoke ROS overproduction, likely due to high TrxR abundance at translational and transcriptional levels fostered by GSH [51, 52]. Overall, the multi-layered anti-oxidant system in tumor cells prompts a multi-targeted approach to deal with clinical chemo/radioresistance.

The recent progress in cancer radiotherapy is based on the stepwise implementation of image-guided and intensity-modulated radiation beams that deliver a shaped dose distribution tailored to tumor anatomy [53]. However, up to 30% of locally advanced cancers show unsatisfactory down-staging even after applying a simultaneous integrated boost that nowadays represents the most intensified radiation approach to improve local control [54]. Further dose escalation would compromise clinical safety, and thus overcoming radioresistance by available pharmaceuticals is an urgent need and necessity in order to address poor outcome in high-risk patients. Our preclinical 4T1 and EMT6 tumor models suggest that an increased local radioresponse is feasible, once the antioxidant defense systems are targeted by specific inhibitors at the cost of marginal if any adverse effects. As outlined above (Supplementary Figure 1), our understanding of inflammatory and ROS-mediated pathways in cancer is evolving, and opens novel possibilities to revisit established drugs with yet uncovered radiosensitizing properties. With this concept in mind, our research program at Radiotherapy Department (UZ Brussels) is making major efforts to re-examine metformin (manuscript in preparation) and AF for radiotherapeutic applications, and the present set of data emphasizes a novel way to exploit the known molecular target TrxR for radiosensitizing purposes. The next pre-clinical steps could be validation of AF-induced radiosensitization in other tumor models and for fractionated radiation.

In conclusion, the anti-arthritic drug AF reveals radiosensitizing properties through targeting TrxR and resulting ROS overproduction, a common mechanism conferring its cytotoxic, antitumor and chemosensitizing effects. Our findings illuminate the redox TrxR/Trx system in cancer cells as an exploitable radiobiological target, and warrant further experimental and clinical approval for AF in combination with radiotherapy.

## MATERIALS AND METHODS

### Cell lines and chemicals

Murine mammary adenocarcinoma EMT-6 cells were kindly provided by Dr. Edith Lord (University of Rochester, Cancer Center, New York) and 4T1 cells were obtained from ATCC (American Type Culture Collection) respectively. All experiments were performed in RPMI 1640 medium (Thermo Fisher, Belgium) supplemented with 10% bovine calf serum (Greiner Bio-one, Belgium). Chemicals were obtained from Sigma-Aldrich (Antwerp, Belgium) unless otherwise stated.

### Treatments

EMT-6 and 4T1 cultures were grown to confluence and treated with AF (2 h) and/or BSO (16 h) at indicated concentrations. The ROS scavenger N-acetyl cysteine (NAC) was added at 10 mM to cultures both 1 h prior and during treatment with AF. Afterwards, cultures were used for further analysis as described below.

### Cytotoxicity and MTT assay

A total of 5000 cells were plated in 100 μl medium in 96-well plates and allowed to adhere for 24 h. AF was added at indicated concentrations for 2 h, and cultures were rinsed with fresh medium and re-incubated for additional 24 h. Next, 10 μl of the MTT reagent (5 mg/ml) was added for 3 h, and 150 μl of DMSO was admixed to dissolve the formazan crystals. Absorbance was measured at a wavelength of 540 nm by using a spectrophotometer (Molecular Devices, Sunnyvale, CA, USA). Cell viability was determined by dividing the absorbance values of treated cells to that of untreated (control) cells.

### Radiation and clonogenic assay

Clonogenic assay was performed as reported in detail elsewhere [22]. Briefly, confluent cultures in 6-well plates were treated with AF and/or BSO and irradiated at a dose rate of 2 Gy/min on a 6 MV Linac (Elekta, Crowley, UK). To induce hypoxia, treated cultures were placed in a tissue-mimetic culture system (TMCS) for 45 min at 37°C in order to metabolically consume oxygen [22]. Cells were then irradiated at indicated doses, trypsinized and reseeded for colony formation. After 8 days, cultures were fixed with crystal violet and colonies (> 50 cells) were counted. Survival fractions (SF) were fitted to the linear quadratic model using GraphPad Prism 5 software (GraphPad Prism Software Inc., La Jolla, CA, USA). Radiosensitization was expressed as an enhancement ratio determined at a SF of 0.1.

### Apoptotic assay

Apoptosis was analyzed by using the double staining with the lipophilic dye Annexin V and 7-amino actinomycin D (7-AAD) (Abcam, Cambridge, UK). Briefly, 0.5 × 10^6^ cells were treated with AF for 2 h, harvested by trypsin, washed twice with PBS and resuspended in 100 μl. Thereafter, 2.5 μl Annexin V-FITC and 5 μl 7-AAD were added to the cell suspensions and incubated for 20 min at room temperature in the dark. Early apoptotic cells (Annexin V-positive, 7-AAD-negative), necrotic/late apoptotic cells (double-positive), and living cells (double-negative) were determined by flow cytometry (BD LSR Fortsessa, BD Bioscience, Franklin Lakes, USA).

### Double-strand DNA breaks

DNA damage following irradiation at 8 Gy was determined by the extent of phosphorylation of the histone protein γH2AX. Cells were treated with AF (7.5 μM) for 2 h, fixed in a buffer (Miltenyi biotec, Leiden, Netherlands) for 15 min at room temperature and permeabilized for 20 min in 90% methanol at −20°C. Next, cells were incubated with 0.1 μg γH2AX antibody (Abcam, Cambridge, UK) for 40 min at 4°C and analyzed by flow cytometry.

### ROS production

The intracellular level of ROS was detected using 5-(6)-chloromethyl-2′,7′-dichlorodihydro-fluorescein diacetate (CM-H*^2^*DCFDA), an oxidation-sensitive fluorescent probe (Abcam, Cambridge, UK). Briefly, cells were treated with AF for 2 h, stained with 5 μM CM-H*^2^*DCFDA at 37°C for 30 min and analyzed by flow cytometry.

### TrxR activity

TrxR activity was measured by using a commercial kit (Sigma-Aldrich, Antwerp, Belgium) according to the manufacturer's instructions. In this assay, TrxR catalyzes the reduction of 5, 5-dithiobis (2-nitrobenzoic) acid (DTNB) to 5-thio-2-nitrobenzoic acid, which generates a strong yellow color. Briefly, cells were treated with AF, lysed with CelLytic Buffer (Sigma-Aldrich, Antwerp, Belgium) and disrupted by sonication for 1min. Afterwards, 180 μl of TE buffer containing DTNB and NADPH was added. The linear increase in absorbance at 412 nm was measured during 30 min using a spectrophotometer (Molecular Devices, Sunnyvale, CA, USA). TrxR activity was calculated as a percentage of enzyme activity to that of DMSO-treated samples.

### GSH assay

GSH levels were measured with a commercial GSH assay kit (Sanbio, Belgium). Briefly, cells were treated with BSO, washed twice with PBS and resuspended in cold MES buffer. Next, cells were lysed by sonication for 1 min, centrifuged at 10000 rpm for 15 min at 4°C and finally deproteinized by phosphoric acid. Afterwards, 50 μl of the collected supernatant was added to 150 μl assay cocktail, and absorbance was measured at 405 nm using a spectrophotometer during 30 minutes with five-minute intervals.

### Oxygen consumption rates

Oxygen consumption rates were determined using a Seahorse XF96 analyzer (Seahorse Biosciences, North Billerica, MA, USA) as previously reported [55]. Briefly, 1.5 × 10^5^ cells were seeded in 96-well plates and 24 h later were treated with AF for 2 h. Afterwards, cultures were equilibrated in unbuffered DMEM with glutamine and glucose at 37°C in a CO*^2^*-free incubator and processed for measurements. To extract detailed information on the electron transport chain in mitochondria, the standard sequence of specific inhibitors consisting of oligomycin, FCCP, rotenone and antimycin A was used.

### Mitochondrial membrane potential

Mitochondrial membrane potential was measured using a potential-dependent positively-charged red-orange dye tetramethylrhodamine ethyl ester (Abcam, Cambridge, UK), which accumulates in active mitochondria due to their relative negative charge. Briefly, after treatment with AF for 2 h, cells were stained with 400 nM dye at 37°C for 30 min and analyzed by flow cytometry.

### Mouse tumor model

BALB/c mice were inoculated intramuscularly into the left hind limb with 4T1 or EMT6 cells (0.5 × 10^6^) and 4 days later randomized with 6 mice/group, AF (3 mg/kg) and/or BSO (25 mg/kg) were administrated subcutaneously from day 6 to 10 and day 13 to 17. Tumors were irradiated with 15 Gy on day 5 at a dose rate of 2 Gy/min on a 6 MV Linac (Elekta, Crowley, UK). The tumor volume was calculated using the formula V = (L * W^2^) * 0.5, where V = volume, L = length, and W = width.

### Statistics

All assays were repeated at least three times. A one-way ANOVA followed by a Bonferonni's multiple comparison tests was performed using GraphPad Prism 5. Data are expressed as mean with corresponding standard deviations. The number of asterisks in the figures indicates the level of statistical significance as follow: *p < 0.05, **p < 0.01, ***p < 0.001, ****p < 0.0001.

## SUPPLEMENTARY MATERIALS FIGURES


