# Supplementary material for: Auranofin radiosensitizes tumor cells through targeting thioredoxin reductase and resulting overproduction of reactive oxygen species
Source: Oncotarget. 2017 Mar 10;8(22):35728–42. doi: 10.18632/oncotarget.16113 (PMC5482612; doi:10.18632/oncotarget.16113)
Supplement: Supplementary file 1 [file oncotarget-08-35728-s001.pdf]

Auranofin radiosensitizes tumor cells through targeting thioredoxin reductase and resulting overproduction of reactive oxygen species

Supplementary Materials

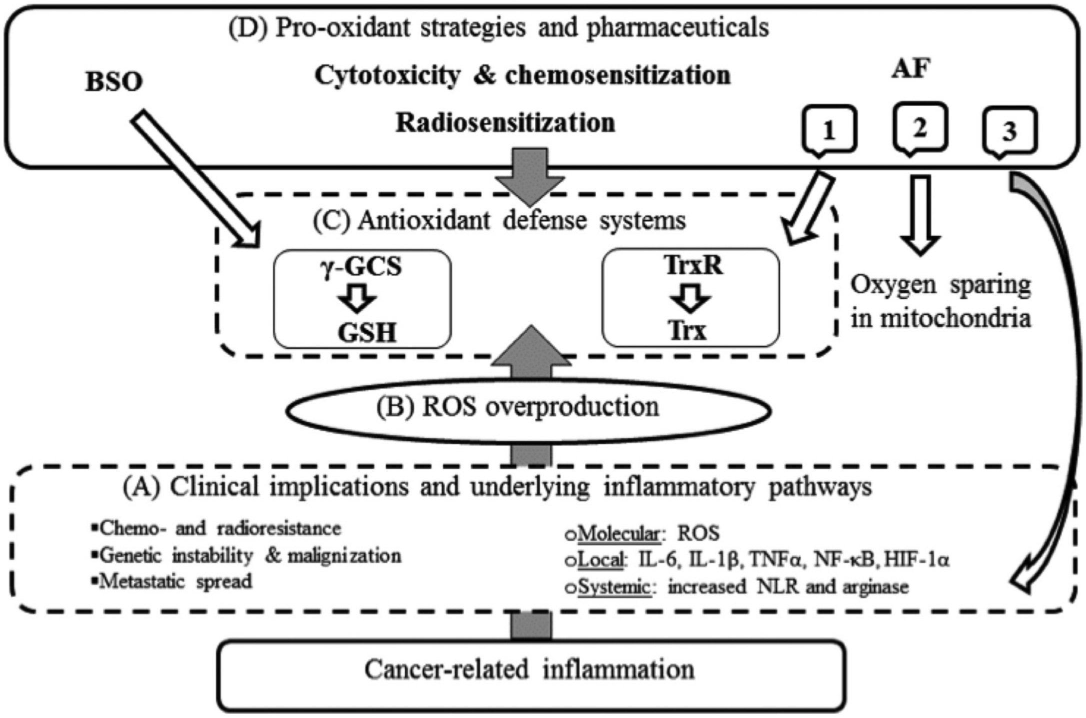

Supplementary Figure 1: Prospects for AF repurposing in cancer radiotherapy. (**A**). The last decade witnessed a tremendous progress in our understanding of ROS, antioxidant defense systems and cancer-related inflammation [1], which is now acknowledged as the 7th hallmark of cancer [2, 3]. Epidemiological studies have linked local inflammation to cancer incidence a long way back but the key masters of complex inflammatory pathways have been unraveled only recently and further linked to clinical implications. An elevated level of neutrophil-to-lymphocyte ratio (NLR) is now established as a prognostic biomarker and reflects a protumor polarization of granulocytes accompanied by a drastic overexpression of arginase. In addition, the monocyte/macrophage lineage within the tumor microenvironment is reprogrammed towards a protumor M2-like phenotype, hence resulting in a skewed polarization of the entire myeloid compartment with a proficient immunosuppressive network. They are a source of inflammatory mediators (IL-6, IL-1β, TNF-α and others) that drive both redox and hypoxic adaptations thus allowing tumor cells to benefit from the chronic oxidative/hypoxic stress. However, the major phenomenon underlying inflammation is the overproduction of ROS (**B**), which occurs along with the adaptive activation of antioxidant defense systems (**C**). This coordinated event directly contributes to cancer progression, as ROS are responsible for the genetic instability that fosters clonal malignization, metastatic spread and eventually acquired chemo- and radioresistance [1]. Due to the constitutively elevated redox defense, anti-inflammatory agents seem to lack efficiency and are considered for chemoprevention rather than for cytoreductive interventions in advanced tumors. On the other hand, pro-oxidant treatments have already been proven to induce strong ROS-mediated cytotoxicity, which explains the antitumor effect of some cytostatics and radiation (**D**). In this context, γ-GCS and TrxR that maintain the redox homeostasis (through GSH and Trx) are of particular interest as cancer targets. Among their pharmacological inhibitors, BSO and AF have been successfully used in preclinical studies and currently undergo clinical evaluation for chemosensitizing purposes. We hypothesize (depicted in D) that AF offers more potential and would expect additional effects: (1) radiosensitization through TrxR inhibition and ROS overproduction, which could be further potentiated by BSO; (2) radiosensitization through spared oxygen, as a result of the oxygen consumption arrest in impaired mitochondria; (3) inhibition of local and/or systemic inflammatory responses, a part of its known anti-arthritic medication, which may be promising for immunocorrection. This study addresses the first two mechanisms with the main focus on local tumor control.

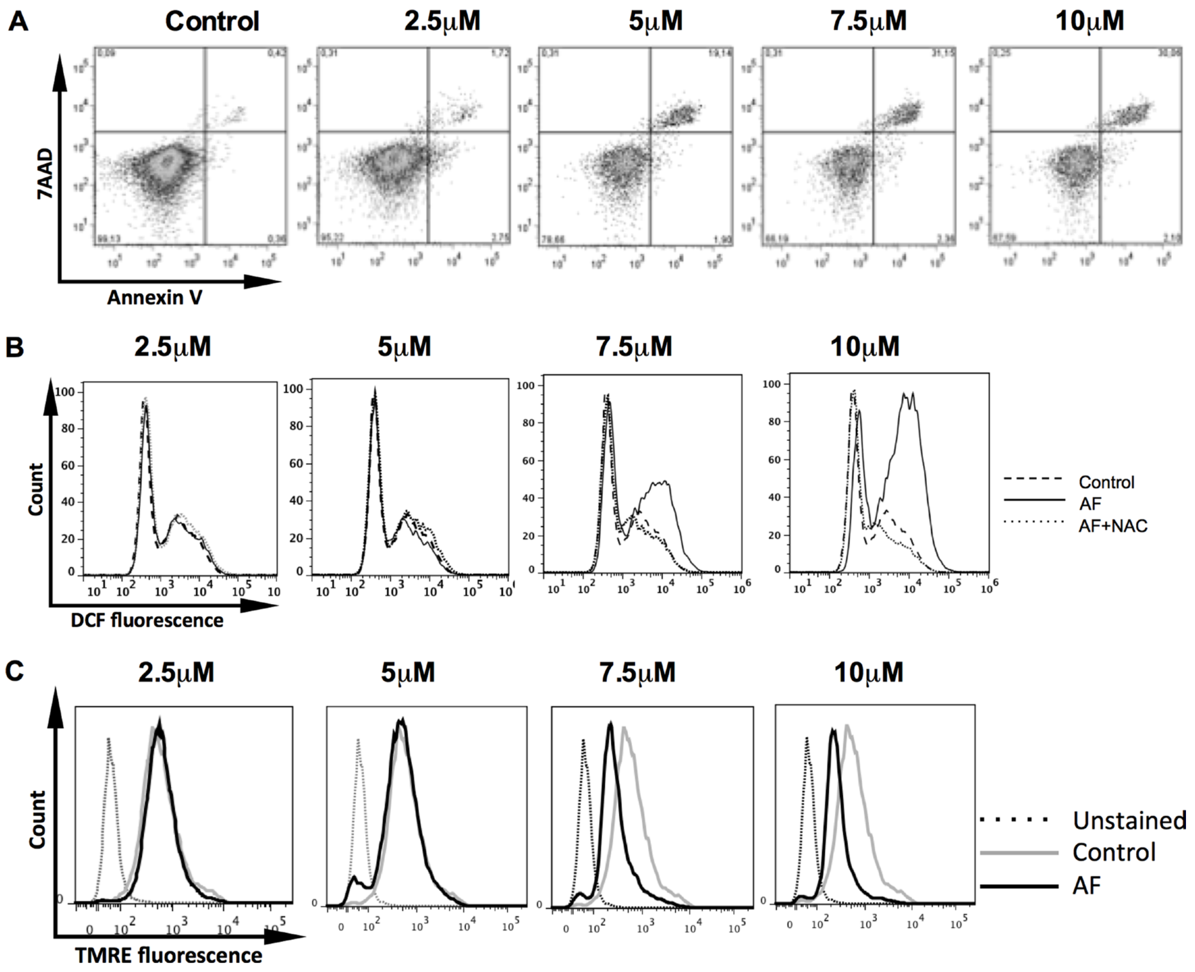

Supplementary Figure 2: Representative flow cytometry plots in EMT6 cells. Cells were treated with AF for 2 h at indicated concentrations. (**A**) Scatter plots of apoptosis. (**B**) Histograms of ROS. (**C**) Histograms of ΔΨm.

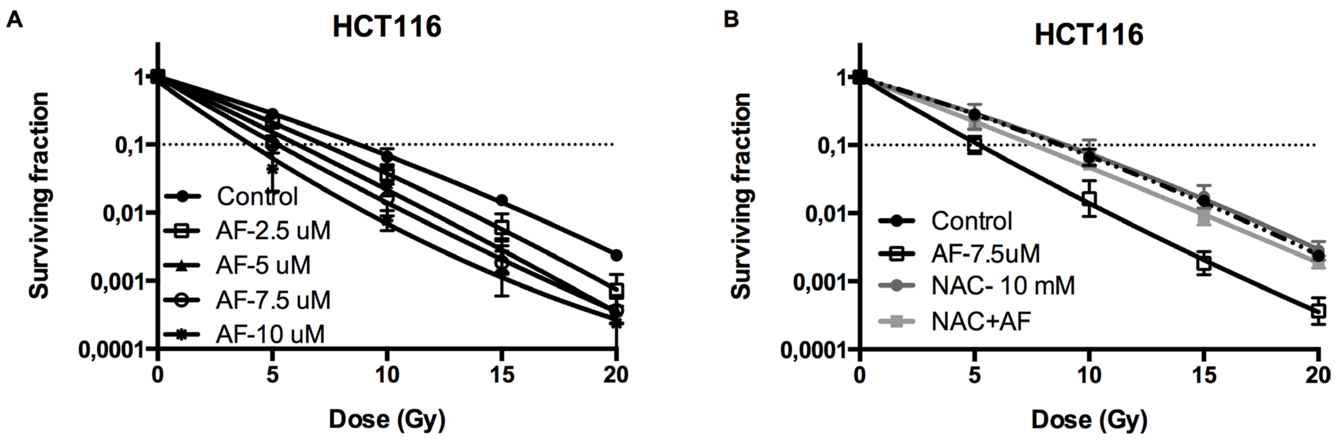

Supplementary Figure 3: AF radiosensitized hypoxic HCT116 cells. Cells were treated with AF for 30 min at indicated concentrations, while NAC (10 mM) was added 1 h prior and during AF treatment. To assess hypoxic radiosensitivity, cells were irradiated in a metabolic hypoxia model. (**A**) Radiosensitizing effect of AF was assessed by colony formation assay. (**B**) Counteracting effect of the ROS scavenger NAC.

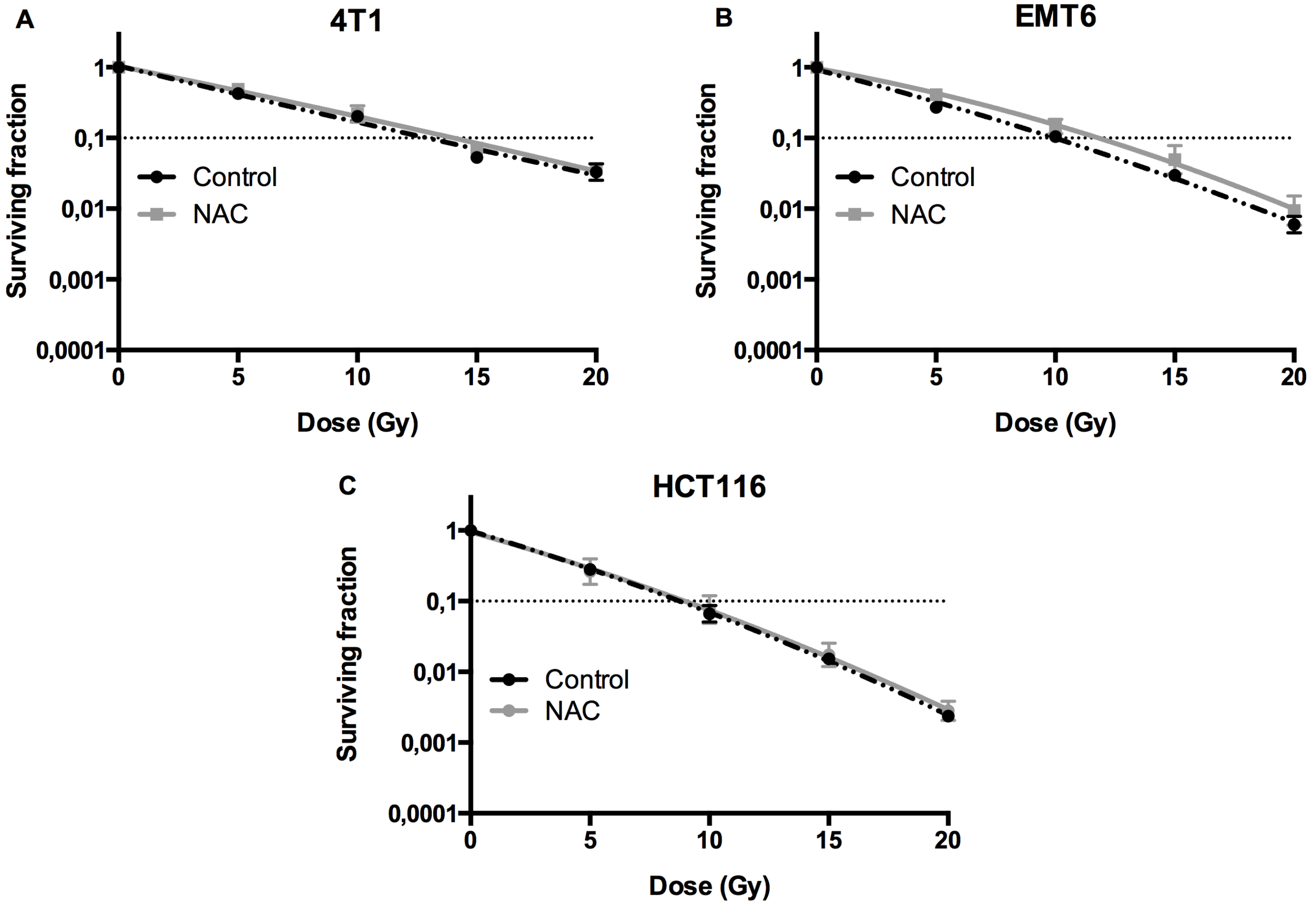

Supplementary Figure 4: No impact of NAC on hypoxic radioresponse of tumor cells. Cells were treated with NAC for 1 h at 10 mM, afterwards irradiated in hypoxia. The survival fraction of cells was assessed by colony formation assay. Survival curves of (**A**) 4T1, (**B**) EMT6, and (**C**) HCT116 cells.

REFERENCES

1. Trachootham D, Alexandre J, Huang P. Targeting cancer cells by ROS-mediated mechanisms: a radical therapeutic approach? Nat Rev Drug Discov. 2009; 8:579–91.
2. Colotta F, Allavena P, Sica A, Garlanda C, Mantovani A. Cancer-related inflammation, the seventh hallmark of cancer: links to genetic instability. Carcinogenesis. 2009; 30:1073–81.
3. Senovilla, Vacchelli E, Galon J, Adjemian S, Eggermont A, Frifman WH, Sautes-Fridman C, Ma Y, Tartour E, Zitvogel L, Kroemer G, Galluzzi L. Trial watch: Prognostic and predictive value of the immune infiltrate in cancer. Oncoimmunology. 2012; 1:1323–1343.
